# Supplementary material for: Young People and the Future: School Students’ Concerns and Hopes for the Future after One Year of COVID-19 in Austria—Findings of a Mixed-Methods Pilot Study
Source: Healthcare (Basel). 2023 Aug 9;11(16):2242. doi: 10.3390/healthcare11162242 (PMC10454506; doi:10.3390/healthcare11162242)
Supplement: Supplementary file 1 [file healthcare-11-02242-s001.zip › healthcare-2488043-supplementary.pdf]

Suppl. Table S1. Study sample characteristics (*N* = 500)

**Variable**	***n***	**%**
Gender		
Female	250	50.0
Male	250	50.0
Migration background		
No	346	69.2
Yes	154	30.8
Region		
Eastern Austria	277	55.4
Southern Austria	100	20.0
Western Austria	123	24.6
School		
College for higher vocational education	238	47.6
Academic secondary school	222	44.4
School for intermediate vocational education	16	3.2
Vocational school	15	3.0
Middle school	5	1.0
Polytechnical school	4	0.8

Note: The NUTS 1 classification (Nomenclature of territorial units for statistics) was used for the classification of major socio-economic regions of Austria (Eastern Austria: Burgenland, Lower Austria, Vienna; Southern Austria: Carinthia, Syria; Western Austria: Upper Austria, Salzburg, Tyrol, Vorarlberg)

Suppl. Table S2. The greatest concerns about the future. The percentages of respondents reporting each main category (in bold) and subcategory of concerns about the future that emerged from the data for question 1: "What is your greatest concern when you think about the future?".

Categories	*n*	%
**School-related concerns about the future**	**135**	**27.0%**
Failing at graduation	66	13.2%
Academic performance	55	11.0%
General mentions related to school	13	2.6%
School organization	8	1.6%
**Pandemic-related concerns about the future**	**95**	**19.0%**
Further development of the pandemic	43	8.6%
Continuation of restrictions	32	6.4%
No return to normality	26	5.2%
Vaccination	8	1.6%
**Concerns about a lack of locus of control in the future**	**81**	**16.2%**
Fear of failure	56	11.2%
Uncertainties/Fears	14	2.8%
Pessimistic outlook	9	1.8%
Lack of life plans	8	1.6%
Lack of self-determination	2	0.4%
**Work-related concerns about the future**	**81**	**16.2%**
Finding a good job	34	6.8%
Finding a job	28	5.6%
Uncertainties regarding further career	16	3.2%
General mentions related to work	7	1.4%
**Concerns about the future related to physical and mental health**	**64**	**12.8%**
Mental health	31	6.2%
Fear to become unhappy	18	3.6%
Fear of disease	13	2.6%
Well-being of others	7	1.4%
**Concerns about the future related to social relationships**	**47**	**9.4%**
Fear of being alone	37	7.4%
Family conflicts	7	1.4%
Relationship	6	1.2%
Starting a family	3	0.6%
**Concerns about the future regarding further education**	**44**	**8.8%**
Uncertainties regarding education path	17	3.4%
Achievement on the education path	11	2.2%
Access opportunities to further education	10	2.0%
General mentions related to studying	6	1.2%
**Concerns about the future related to social and political developments**	**39**	**7.8%**
Climate crisis	16	3.2%
Economic/Financial crisis	14	2.8%
Political situation	14	2.8%
Pandemics/Other crises	4	0.8%

**Concerns about the future related to the financial situation**	**34**	**6.8%**
**Other concerns about the future**	**11**	**2.2%**
**No concerns about the future/"I don't know"**	**23**	**4.6%**

Note: The percentages of the main categories may differ from the sum of the percentages in the individual subcategories because it may be that a respondent reported experiences in several subcategories (e.g., academic performance and school organization) within one main category (e.g., school-related concerns).

Suppl. Table S3. The greatest hopes for the future. The percentages of respondents reporting each main category (in bold) and subcategory of concerns about the hopes for the future that emerged from the data for question 2: " What do you hope for most when you think about the future?".

Categories	*n*	%
**Pandemic-related hopes for the future**	**181**	**36.2%**
Return to normality	92	18.4%
End of restrictions	69	13.8%
End of the pandemic	55	11.0%
Vaccination	7	1.4%
Reassurance	4	0.8%
**Hopes for the future regarding major life goals**	**142**	**28.4%**
Life satisfaction	83	16.6%
Self-actualization	40	8.0%
Autonomy	14	2.8%
Personal strength	11	2.2%
Life full of experiences	5	1.0%
**Hopes for the future relating to social relationships**	**95**	**19.0%**
Contacts with friends	52	10.4%
Starting a family	30	6.0%
Good relationship	17	3.4%
Less conflicts in family of origin	12	2.4%
**Health-related hopes for the future**	**89**	**17.8%**
Well-being	34	6,8%
Physical health	32	6.4%
Well-being of others	16	3,2%
Fitness	11	2.2%
Mental health	6	1.2%
**School-related hopes for the future**	**71**	**14.2%**
Graduation	34	6.8%
Academic performance	18	3.6%
School organization	18	3.6%
Less stress at school	8	1.6%
**Hopes for the future related to lifestyle**	**70**	**14.0%**
Secure financial situation	38	7.6%
Travel abroad	17	3.4%
Living situation	9	1.8%
Stability	6	1.2%
Driving license/Car	6	1.2%
**Work-related hopes for the future**	**54**	**10.8%**
Find a good job	43	8.6%
Find a job	6	1.2%
Successful career	5	1.0%
**Hopes for the future regarding further education**	**34**	**6.8%**
General mentions of further education	17	3.4%
Vocational aspirations	12	2.4%

Academic success	5	1.0%
**Hopes for the future related to social and political developments**	**29**	**5.8%**
Social developments	16	3.2%
Political developments	6	1.2%
Recognition of climate change	6	1.2%
Economic developments	2	0.4%
No other crisis	2	0.4%
**Other hopes for the future**	**11**	**2,2%**
**No hopes for the future/"I don't know"**	**9**	**1.8%**

Note: The percentages of the main categories may differ from the sum of the percentages in the individual subcategories because it may be that a respondent reported experiences in several subcategories (e.g., graduation and academic performance) within one main category (e.g., school-related hopes).

Suppl. Table S4. Proportion of female and male students differing in reportings in main or subcategories of 1) Concerns about the future and 2) Hopes for the future (*N* = 500)

Category		Gender		Statistics
		Female (*n* = 250)	Male (*n* = 250)	
1) Concerns about the future:				
No return to normality	%	7.6	2.8	$\chi2(1) = 5.842$;
	n	19	7	$p = 0.016$
Access opportunities to further education	%	3.6	0.4	$p = 0.020$
	n	9	1	
**Lack of locus of control**	%	21.6	10.8	$\chi2(1) = 10.740$;
	n	54	27	$p = 0.001$
Fear of failure	%	15.6	6.8	$\chi2(1) = 9.733$;
	n	39	17	$p = 0.002$
**Physical and mental health**	%	17.2	8.4	$\chi2(1) = 8.673$;
	n	43	21	$p = 0.003$
Mental health	%	8.8	3.6	$\chi2(1) = 5.812$;
	n	22	9	$p = 0.016$
Fear to become unhappy	%	6.0	1.2	$p = 0.007$
	n	15	3	
**Social and political developments**	%	4.8	10.8	$\chi2(1) = 6.257$;
	n	12	27	$p = 0.012$
No concerns about the future	%	1.6	5.6	$p = 0.028$
	n	4	14	
2) Hopes for the future:				
Life as before the pandemic	%	22.8	14.0	$\chi2(1) = 6.447$;
	n	57	35	$p = 0.011$
**Major life goals**	%	33.6	23.2	$\chi2(1) = 6.649$;
	n	84	58	$p = 0.010$
Life satisfaction	%	22.8	10.4	$\chi2(1) = 13.883$;
	n	57	26	$p < 0.001$

p: *p*-values (2-tailed); $\chi2$: Chi-squared-test. The main categories are in bold.

Suppl. Table S5. Proportion of students with and without migration background differing in reportings in main or subcategories of 1) Concerns about the future and 2) Hopes for the future ($N = 500$)

Category		Migration background yes ($n = 154$)	Migration background no ($n = 346$)	Statistics
1) Concerns about the future:				
**Pandemic-related concerns about the future**	%	13.6	21.4	$\chi2(1) = 4.160$;
	n	21	74	$p = 0.041$
**Lack of locus of control**	%	23.4	13.0	$\chi2(1) = 8.443$;
	n	36	45	$p = 0.004$
Fear of failure	%	18.8	7.8	$\chi2(1) = 13.031$;
	n	29	27	$p < 0.001$
**Social and political developments**	%	3.9	9.5	$\chi2(1) = 4.716$;
	n	6	33	$p = 0.030$
2) Hopes for the future:				
**Pandemic-related hopes for the future**	%	29.9	39.0	$\chi2(1) = 3.861$;
	n	46	135	$p = 0.049$
End of restrictions	%	7.1	16.8	$\chi2(1) = 8.291$;
	n	11	58	$p = 0.004$
Well-being of others	%	6.5	1.7	$\chi2(1) = 7.793$;
	n	10	6	$p = 0.005$

p: *p*-values (2-tailed); χ2: Chi-squared-test. The main categories are in bold.

Suppl. Table S6. Proportion of students with and without psychological distress differing in reportings in main or subcategories of 1) Concerns about the future and 2) Hopes for the future ($N = 500$)

Category		Psychological distress yes ($n = 310$)	Psychological distress no ($n = 190$)	Statistics
1) Concerns about the future:				
Vaccination	%	0.3	3.7	$p = 0.006$
	n	1	7	
**School-related concerns about the future**	%	31.6	19.5	$\chi2(1) = 8.807$;
	n	98	37	$p = 0.003$
Failing at graduation	%	16.5	7.9	$\chi2(1) = 7.528$;
	n	51	15	$p = 0.006$
Fear of failure	%	13.5	7.4	$\chi2(1) = 4.524$;
	n	42	14	$p = 0.033$
**Physical and mental health**	%	15.8	7.9	$\chi2(1) = 6.606$;
	n	49	15	$p = 0.010$
Mental health	%	8.1	3.2	$\chi2(1) = 4.877$;
	n	25	6	$p = 0.027$
**No concerns about the future/"I don't know"**	%	2.3	8.4	$\chi2(1) = 10.196$;
	n	7	16	$p = 0.001$
No concerns about the future	%	1.6	6.8	$\chi2(1) = 9.282$;
	n	5	13	$p = 0.002$
2) Hopes for the future:				
**Major life goals**	%	31.6	23.2	$\chi2(1) = 4.141$;
	n	98	44	$p = 0.042$
Life satisfaction	%	19.4	12.1	$\chi2(1) = 4.472$;
	n	60	23	$p = 0.034$
Social developments	%	1.9	5.3	$\chi2(1) = 4.211$;
	n	6	10	$p = 0.040$

p: *p*-values (2-tailed); χ2: Chi-squared-test. The main categories are in bold.
